# Kind Words and Suggestions

**Published:** 1873-05

**Authors:** 


*Kind Words and Suggestions.*

In the April number of THE SOUTHERN MEDICAL RECORD, I see a letter from Dr. J. A. Hanks, which has the ring of the true metal. His words are kind and true—his suggestions sound and good. Taken collectively, they are a high compliment both to his head and heart. That his advice may be followed implicitly by every subscriber to the RECORD I sincerely trust.

Yours, sincerely, ALBAN S. PAYNE, M.D.

These kind words from our Virginia associate are appreciated highly. The RECORD, upheld and supported by such names as those of our large list of associates, can "do and dare" almost anything. Go to work, friends. The RECORD will increase in size and power just in proportion to the energy put forth by its friends. Its future is in their hands. While we hold the helm, will they not give the power?

